# Perioperative iron deficiency anaemia

**DOI:** 10.1016/j.bjae.2023.06.001

**Published:** 2023-07-06

**Authors:** A. Shah, A. Acheson, R.C.F. Sinclair

**Affiliations:** 1 University of Oxford, Oxford, UK; 2 University of Nottingham, Nottingham, UK; 3 Royal Victoria Infirmary, Newcastle Upon Tyne Hospitals NHS Trust, Newcastle, UK

**Keywords:** anaemia, iron deficiency, perioperative care

**Learning objectives**

By reading this article, you should be able to:

• Describe iron physiology in health and in disease states relevant to perioperative medicine.
• Interpret laboratory tests, in particular markers of iron status, in the diagnostic work-up of perioperative anaemia.
• Outline the prevalence of iron deficiency anaemia in various groups of surgical patients.
• Discuss the benefits and risks of treatments for perioperative anaemia including iron therapy, erythropoiesis-stimulating agents and blood transfusion.
• Explain recent guidelines and clinical trials on the management of perioperative anaemia.

**Key points**

• Iron deficiency is the most common cause of anaemia, affecting at least 1.2 billion people worldwide.
• Iron is essential for haemoglobin synthesis, cell growth and differentiation, oxygen sensing, muscle energetics and cellular immunity.
• Systemic iron homeostasis is finely regulated by hepcidin.
• Pre- and postoperative anaemia affects nearly all groups of patients and is an independent risk factor for poor clinical outcomes after both elective and non-elective surgery.
• Perioperative iron deficiency anaemia is commonly treated with oral or i.v. iron with some evidence of improved clinical outcomes. Ongoing research will provide further evidence on the use of erythropoiesis-stimulating agents.

Iron deficiency affects ∼2 billion people worldwide.^1^ It is the commonest cause of anaemia and becomes more important when we consider functional iron deficiency or iron sequestration secondary to inflammation.

This review provides an update on the epidemiology, underlying mechanisms, clinical implications and management of perioperative iron deficiency anaemia in patients undergoing elective and emergency surgery. The detection and management of perioperative anaemia is one of the three key pillars of patient blood management, along with minimising blood loss and bleeding, and optimising the patient's physiological reserve of anaemia. The latter two are beyond the scope of this article and are detailed elsewhere.^2^

## Underlying mechanisms

The aetiology of perioperative iron deficiency anaemia is multifactorial and can be secondary to: (i) pre-existing nutritional deficiency, underlying comorbidities (e.g. cancer), or both; (ii) anaemia of inflammation; and (iii) blood loss from the surgical procedure itself. Many of these factors coexist in clinical practice. Other causes can be summarised as being attributable to:

• Reduced absorption—bariatric surgery, coeliac disease, gastritis, drugs (e.g. proton pump inhibitors)
• Reduced intake—eating disorders, vegetarians/vegans
• Increased requirements—infants, adolescents, athletes, pregnancy, blood donors
• Chronic blood loss—gastrointestinal tumours, hookworm infestation, abnormal uterine bleeding.^1^^,^^3^

### In health

Iron is essential for haemoglobin (Hb) synthesis. Humans normally synthesise at least 2 million erythrocytes per second. Each mature red blood cell (RBC) contains 280 million molecules of Hb and each of the four globin subunits contains one iron atom in haem, resulting in the total iron flux required to maintain erythropoiesis being 2–3 × 10^15^ atoms per second in the adult human.^4^

Total body iron content is ∼3–4 g, of which 1–2 mg is lost every day, and a further 1 mg is approximately lost monthly during menstruation. Humans are unable to excrete iron actively and have therefore developed finely tuned regulatory mechanisms to control the amount of dietary iron intake, cellular iron uptake, bodily distribution and export.^5^ Dietary iron must pass through absorptive enterocytes to enter the circulation. Haem iron is the most effectively absorbed. Inorganic non-haem ferric iron must be reduced to the soluble ferrous (Fe^2+^) iron by brush border ferrireductase before it can be absorbed.

**Fig 1:**
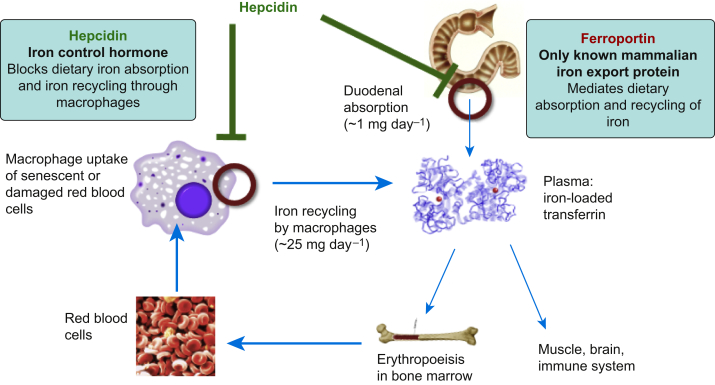
Hepcidin–ferroportin interaction and major systemic iron pathways.

Systemic iron homeostasis (Fig. 1) is finely regulated by hepcidin, which is predominantly produced in the liver.^5^ Hepcidin expression results in degradation of ferroportin, the only known mammalian exporter of iron, which blocks the release of iron from macrophages and duodenal enterocytes and subsequently reduces iron availability.^5^ Hepcidin is upregulated in the presence of inflammation and high circulating concentrations of transferrin-bound iron, labile iron, or both. Conversely, hepcidin concentrations are decreased in iron deficiency, hypoxia, during blood loss and increased erythropoietic activity (via erythroferrone).^5^ Genetic loss of hepcidin control leads to disorders of iron overload such as haemochromatosis, thalassaemia syndromes (α and β) and congenital dyserythropoietic anaemia.^1^

### Inflammation

Stressors such as an acute illness, infection or a surgical stress response trigger a host immune and inflammatory response resulting in profound changes in iron trafficking. The proinflammatory cytokine, interleukin (IL)-6, seems to play the most important role in developing anaemia.^6^ IL-6 upregulates hepcidin, which in turn blocks duodenal iron absorption and causes iron retention in macrophages (‘hepcidin block’) leading to a state of iron-restricted erythropoiesis. This can also be considered to be part of the evolutionary response (‘nutritional immunity’) to limit free (or non-transferrin-bound iron) for invading pathogens, particularly iron-dependent extracellular bacteria (e.g. *Staphylococcus aureus*, *Escherichia coli*, *Klebsiella pneumonia*) that could otherwise cause overwhelming infection.^7^

The cytokines IL-1, IL-6, IL-10 and tumour necrosis factor (TNF)-α also promote iron sequestration into macrophages via transferrin receptor-mediated endocytosis. Inflammation also reduces production of erythropoietin and its efficacy through IL-1 and IL-6.^6^ Recovery from inflammation has been shown to lower hepcidin and IL-6 levels, leading to increased release of iron for Hb synthesis.

### Haemoglobin-independent effects of iron

Iron is essential for many other physiological processes such as cell growth and differentiation, neurotransmission, host defence and cardiopulmonary function.^7^, ^8^, ^9^ Many cellular proteins require iron to function. Examples include components of the mitochondrial electron transport chain. Enzymes involved in DNA metabolism, such as ribonucleotide reductase, DNA primase and DNA helicase bind iron.^5^ The ATPase ABCE1, which is needed for messenger RNA translation, also binds iron. Iron also plays a critical role in oxygen sensing as part of the hypoxia inducible factor (HIF) pathway. The degradation of HIFs, especially HIF-2α by prolyl hydroxylases, is dependent on both iron and oxygen, and studies in healthy volunteers have demonstrated that iron depletion augments the pulmonary hypertensive response to hypoxia, whilst iron loading attenuates this response.^10^ Iron is also essential for immunity. Both iron deficiency and iron overload are associated with increased risk of infection.^11^ Recent work has demonstrated that hypoferraemia impairs the adaptive immune response (T and B cells) to infection and vaccination.^9^

## Investigations

Assessment of perioperative anaemia necessitates a careful history, clinical examination and analysis of a ‘battery’ of tests. Current consensus is to use a Hb threshold of <130 g L^−1^ to define anaemia in both sexes.^12^ A summary of the different types of iron deficiency, expected laboratory findings and potential treatment strategies is displayed in Table 1.

**Table 1:** Definitions, laboratory characteristics and potential treatment strategies of the various stages of iron deficiency. CHr, reticulocyte haemoglobin content; CRP, C-reactive protein; eGFR, estimated glomerular filtration rate; Hb, haemoglobin; i.v., intravenous; Tsat, transferrin saturation.

Iron status	Definition	Laboratory findings	Iron therapy strategies
True/absolute iron deficiency	Depletion of body iron stores, which are inadequate to maintain erythropoiesis	Ferritin <30 μg L^−1^ or Tsat <20% and/or CHr<30 pg, Hb >130 g L^−1^, low hepcidin	Oral iron, or i.v. iron if poorly tolerated
Iron-deficiency anaemia	Reduced Hb and erythrocytes because of insufficient iron vailability	Hb <130 g L^−1^, ferritin <30 μg L^−1^ or Tsat <20% and/or CHr <30 pg, low hepcidin	Oral iron, i.v. iron if poorly tolerated or rapid correction required
Iron sequestration/iron-restricted erythropoiesis	Insufficient mobilisation of iron stores because of increased demands, despite adequate iron stores	Ferritin >100 μg L^−1^ and Tsat <20%, or CHr ≥30 pg, or both Possibly increased CRP Variable hepcidin	Erythropoietin and i.v. iron, consider i.v. iron alone if Hb is normal
Iron deficiency in the presence of inflammation	Reduced supply (functional) or availability (absolute) of iron to meet erythropoietic requirements	Ferritin 30–100 μg L^−1^, Tsat <20%, CRP >5 mg L^−1^ or GFR <60 ml min^−1^ Increased hepcidin	I.V. iron, consider erythropoietin if response to i.v. iron alone is inadequate

A detailed review of the different indicators of iron status can be found elsewhere but in brief, serum ferritin is currently the most commonly used indicator of iron deficiency.^3^ However, ferritin is an acute phase reactant and may be increased in inflammatory states (e.g. surgery), so normal ferritin values do not exclude iron deficiency. Measurement of C-reactive protein is recommended to aid interpretation of serum ferritin concentrations. Other useful tests include transferrin saturation (Tsat)—a marker of the amount of iron bound to transferrin and reticulocyte haemoglobin content (CHr)—a measure of the amount of iron available for Hb production in the bone marrow. The use of newer markers of iron status, such as hepcidin, soluble transferrin status and erythroferrone, is an area of active research.

## Epidemiology and clinical implications

### Elective non-cardiac surgery

Preoperative anaemia affects 30–60% of patients and varies according to the type of surgery.^13^^,^^14^ The highest prevalence has been reported in patients undergoing gynaecological surgery (64%) and colorectal cancer resection (58%), with women being disproportionately affected with a prevalence of 68.9%.^14^ The major cause of anaemia in both these groups was absolute iron deficiency. Among all patients with anaemia, approximately two-thirds have evidence of absolute iron deficiency, 10–15% have iron sequestration and <10% have B12 or folate deficiency.^14^

Preoperative anaemia is associated with an increased risk of blood transfusion, in-hospital complications, delayed hospital discharge and poor recovery.^13^ More recent work has demonstrated that postoperative anaemia in patients who have undergone major abdominal surgery is associated with increased risk of death or disability up to 90 days after surgery, more postoperative complications, increased risk of unplanned ICU admission and longer hospital stays.^15^ Preoperative anaemia may also limit the ability to perform acute normovolaemic haemodilution.

### Cardiac surgery

Preoperative anaemia is also common in cardiac surgery, affecting up to 40% of patients. It is independently associated with an increased risk of blood transfusion, increased mortality and longer hospital stays.^16^ Nearly one in two patients are iron deficient before surgery. This is relevant, particularly for patients with impaired left ventricular function, as treating iron deficiency with i.v. iron in patients with congestive cardiac failure has been shown to improve functional status and reduce cardiovascular-related hospital readmissions and mortality.^17^

### Urgent and emergency surgery

Anaemia around the time of major emergency surgery is very common.^18^ In contrast to planned procedures, there is not a period for preoperative optimisation, especially in view of guidance advocating that surgery be expedited (e.g. for surgical fixation of fractured neck of femur). In patients undergoing emergency laparotomy, more than half have evidence of anaemia before surgery, increasing to 60–80% in the postoperative period. Even ‘mild’ anaemia (Hb 110–126 g L^−1^) is independently associated with increased mortality, prolonged hospitalisation and increased risk of reoperation.^15^ Similar trends have been observed in patients undergoing hip fracture surgery, where the aetiology is multifactorial, arising from either chronic disease or blood loss at the time of injury or surgery.^18^ Anaemia on hospital admission is associated with a negative impact on postoperative functional recovery, length of hospital stays and readmission rates.^18^ One study found a linear association between higher Hb concentrations and improved functional status and that anaemia was an independent risk factor for inability to walk on the third postoperative day.^18^

### Obstetrics

In pregnancy, iron deficiency usually results from an imbalance of supply and demand. Approximately 45% of women begin pregnancy with low iron stores.^19^ The incidence of maternal anaemia is around 50% in low- and middle-income countries, largely a combination of nutritional deficiency, infectious diseases and the presence of a variant Hb or a thalassaemia disorder. The prevalence of anaemia ranges from 24% to 46% at the booking or 28-week check in the UK.

Maternal anaemia is an independent risk factor for adverse peripartum and perinatal outcomes include preterm labour, small-for-gestational age babies, low birth weight, increased fetal and neonatal mortality and obstetric haemorrhage.^19^ Data are beginning to emerge on neurocognitive outcomes of children born to women with iron deficiency. Iron deficiency *in utero* (defined as cord blood ferritin <75 ng ml^−1^) is associated with poor memory, altered interactions with caregivers and abnormal neurological reflexes in infants.^19^

### Paediatric surgery

Children aged <5 yrs are especially susceptible to the long-term effects of untreated anaemia. A series of observational studies have shown that preoperative anaemia is independently associated with increased in-hospital mortality, prolonged hospital length of stay and increased need for blood transfusion in children, neonates, or both undergoing surgery.^20^ However, it remains unclear what the best way of identifying and treating anaemia is in this cohort of patients.

## Perioperative treatment strategies

Comprehensive and detailed guidance on the identification and management of perioperative anaemia has recently been published by the Centre for Perioperative Care (CPOC).^3^ Treatment through dedicated preoperative anaemia clinics may be associated with improvements in clinical outcomes and lower transfusion requirements. Treatment time before surgery is correlated with a greater increase in Hb up until 2 months before surgery, after which further time may not increase Hb.^21^

### Enteral iron

Oral iron, in the form of ferrous sulphate or ferrous fumarate, is a cheap and successful way of treating iron deficiency. It is absorbed in the duodenum and jejunum but has a low bioavailability. It is often poorly tolerated by patients because of gastrointestinal side-effects, principally constipation. Correcting whole body iron store depletion requires a course of enteral iron to be taken over several months.

Recent mechanistic and clinical studies on iron kinetics have demonstrated that once a day or alternate day dosing may be better absorbed and tolerated compared with traditional higher doses. A single dose of ferrous sulphate results in a rapid rise in circulating hepcidin, which can remain increased for up to 48 h. Any further oral iron doses will be ineffective because of this ‘hepcidin block’ and risks exposing patients to adverse effects. As a result, ∼40–60 mg iron daily or 80–100 mg iron on alternate days is now recommended by clinical guidelines: this equates to ferrous sulphate/ferrous fumarate 200 mg once daily or 400 mg once daily on alternate days.^12^

Sucrosomial iron is a promising new oral iron-containing carrier. Ferric pyrophosphate is protected by a phospholipid bilayer membrane (sucrosome) creating a complex that can be transported to the duodenal mucosa.^22^ This complex protects iron from the acidic environment of the stomach and the normal hepcidin–ferroportin absorption pathways. It can be tolerated in high doses. Early phase studies, mostly in cardiac surgery, show promising results but larger trials are required.^22^

#### Clinical use

For many planned procedures there is an appropriate treatment window available before surgery to prescribe a course of enteral iron. Enteral iron is more likely to be a successful treatment in the absence of pathologies causing ongoing blood loss (e.g. malignancy, menorrhagia) or inflammation. Certain surgical patients, especially those with cancer, will have a combined anaemia from blood loss, direct effects of the cancer, treatment (e.g. chemotherapy), or both on erythropoiesis, poor nutrition and concomitant inflammation rendering oral iron ineffective. Prescribing oral iron in the emergency perioperative or early postoperative period is also unlikely to replenish iron stores or treat anaemia.

### Intravenous iron

I.V. iron can bypass the ‘hepcidin block’ caused by inflammation and replenish intracellular iron stores. After i.v. iron treatment, Hb increases quickly with 50% of the effect evident at 3 days, 75% at 2 weeks and maximal response after 4 weeks in true iron deficiency anaemia; many patients report accompanying improvements in well-being.^12^^,^^23^ More recent iron formulations, such as ferric carboxymaltose (FCM) and ferric derisomaltose (FDM; previously known as iron isomaltoside), enable delivery of iron to the reticuloendothelial system in a controlled manner, thus limiting the amount of toxic circulating free iron.^19^ I.V. iron results in improvements in Hb concentration, functional performance and quality of life, and reduction of transfusion requirements for many chronic conditions (e.g. inflammatory bowel disease, chronic kidney disease and heart failure).

#### Clinical use

Current guidelines recommend its use in patients who are planned to undergo surgery within 4–6 weeks, cannot tolerate oral iron, or both.^12^ However, a shorter time frame to surgery should not preclude treatment. Clinical trials have shown improvements in Hb with i.v. iron compared with oral iron or placebo, but the effect on reducing perioperative transfusion requirements is inconsistent (Table 2).^24^, ^25^, ^26^, ^27^, ^28^, ^29^, ^30^, ^31^ This may, in part, be explained by heterogeneity in patients studied, interventions delivered and improvements in usual care (or comparator arms) over time. Secondary outcomes in these trials have demonstrated improvements in quality of life, hospital length of stay and fewer readmissions, but confirmatory studies are needed.

**Table 2:** Summary characteristics of key recently completed and ongoing trials on the management of perioperative anaemia. CHr, reticulocyte haemoglobin content; CI, confidence interval; DAH, days at home; EPO, erythropoietin; FCM, ferric carboxymaltose; FDM, ferric derisomaltose; Hb, haemoglobin; HRQoL, health-related quality of life; IDA, iron deficiency anaemia; ISRCTN; International Standard Randomised Controlled Trial Number; IQR, inter-quartile range; i.v., intravenous; MACE, major adverse cardiovascular events; MD, mean difference; NCT, National Clinical Trial; OR, odds ratio; RBC, red blood cell; RR, relative risk; Tsat, transferrin saturation; TXA, tranexamic acid.

Study	Patients and no. randomised	Intervention(s)	Comparator(s)	Key findings
**Completed trials**				
Talboom and colleagues FIT (2023)^27^	Adults (age >18 yrs) with M0 stage colorectal cancer scheduled for elective curative resection and IDA (Hb: <130 g L^−1^ in men, <120 g L^−1^ in women; and Tsat <20%) *n*=202	I.V. FCM 1–2 g	Ferrous fumarate 200 mg three times daily	No difference in normalisation of Hb at day of admission between both groups (RR 1.08; 95% CI: 0.55–2.10) Higher proportion of patients with normalised Hb in the i.v. iron group at later timepoints (49/82 [60%] *vs* 18/88 [21%] at 30 days; RR 2.92; 95% CI: 1.87–4.58)
Lasocki and colleagues HIFIT (2022)^28^	Adults aged >18 yrs old with osteoporotic fractures of upper end of femur and preoperative haemoglobin 9.5–13 g L^−1^ *n*=419	Three comparator arms: i.v. FDM and TXA or i.v. FDM and placebo or i.v. TXA and placebo	Placebo infusions	Compared with double placebo, i.v. FDM and TXA associated with an ∼50% reduction in transfusion requirements (31/103 [30.1%] *vs* 16/104 [15.4%]; RR 0.51; 95% CI: 0.30–0.88) (preprint results)
Richards and colleagues PREVENTT (2020)^26^	Adults (age >18 yrs) identified with anaemia (Hb: <130 g L^−1^ in men, <120 g L^−1^ in women) 10–42 days before major open abdominal surgery *n*=487	I.V. FCM 1000 mg in 100 ml 0.9% saline	I.V. 100 ml 0.9% saline	No difference in primary outcome (composite of blood transfusion and mortality at 30 days) between both groups (RR 1.03; 95% CI: 0.78–1.37) Fewer hospital readmissions in the i.v. iron group 8 weeks after the index operation Hb concentrations were significantly higher at 8 weeks and 6 months after the index operation in patients who received i.v. iron
Spahn and colleagues (2019)^29^	Patients undergoing elective cardiac surgery with anaemia (Hb: <130 g L^−1^ in men, <120 g L^−1^ in women) or isolated iron deficiency (ferritin <100 μg L^−1^) *n*=484	I.V. FCM (20 mg kg^−1^, max 1000 mg), s.c. EPO 40,000 U, s.c. vitamin B12 1 mg, oral folic acid 5 mg, given day before surgery	Equivalent volumes of placebo	Combination treatment resulted in fewer RBC transfusions from a median (IQR) of 1 (0–3) RBC unit in the placebo group to a median (IQR) of 0 (0–2) RBC units in the treatment group (OR 0.7; 95% CI: 0.50–0.98) Patients in the intervention group had higher Hb, reticulocyte and CHr during the first 7 days after surgery
Keeler and colleagues (2017)^31^	Patients with colorectal cancer scheduled to undergo surgery with Hb 1 g dl^−1^ below the WHO definition *n*=116	I.V. FCM, based on Hb and weight, in 250 ml of 0.9% saline	Oral ferrous sulphate 200 mg twice daily until day of surgery	No difference in RBC transfusion requirements during the perioperative period between both groups Higher Hb rise before surgery in the i.v. group compared with oral iron, with fewer patients with anaemia at the time of surgery
Kim and colleagues FAIRY (2017)^25^	Patients with Hb 7–10 g dl^−1^ at 5–7 days after radical gastrectomy *n*=454	I.V. FCM dosed according to body weight	0.9% Saline	Improved Hb response (defined as rise in Hb >2 g dl^−1^ from baseline) at 12 weeks after surgery in the i.v. group (absolute difference 38.2%, 95% CI: 33.6%–42.8%)
Khalafallah and colleagues (2016)^24^	Patients with functional IDA (Hb 70–120 g L^−1^, ferritin <100 μg L^−1^ or Tsat <20%) at day 1 after surgery, after major elective non-cardiac surgery *n*=201	i.v. FCM 1000 mg	Standard care	Statistically significant higher Hb at 4 weeks after surgery in patients who received i.v. iron (MD 7.84 g L^−1^, 95% CI: 3.79–11.9) Improvements in serum iron, Tsat and ferritin also observed at 4 weeks in the i.v. group. Higher role physical SF-36 scores at 4 and 12 weeks in the i.v. iron group
Froessler and colleagues (2016)^30^	Patients expecting abdominal surgery with preoperative IDA (ferritin <300 μg L^−1^, transferrin saturation <25%, Hb <12.0 g dl^−1^ for women, Hb <13.0 g dl^−1^ for men) *n*=72	I.V. FCM 15 mg kg^−1^ up to 1000 mg before surgery and second dose FCM within 2 days of surgery (dose of 0.5 mg per ml of blood loss during surgery)	Usual care: this included all preoperative anaemia treatments offered by usual care team (some patients received i.v. iron)	The trial was stopped early because of safety concerns over the primary endpoint of blood transfusion A lower incidence of transfusion was seen in the intervention group compared with the usual care arm, 12.5% *vs* 32.25% Decreased LOS in i.v. iron group, (7.0 *vs* 9.7 days, *p*=0.026), and higher Hb at 4 weeks (1.9 *vs* 0.9 g dl^−1^, *p*=0.01) when compared with usual care
**Ongoing trials**				
**Study**	**Patients and planned sample size**	**Intervention(s)**	**Comparator(s)**	**Study outcome(s)**
RESULT-HIP (recruiting) ISRCTN 28818784	Adults aged >60 yrs presenting with hip fracture and anaemia, Hb <90 g L^−1^ *n*=1964	Liberal transfusion strategy initiated when Hb ≤90 g L^−1^, maintenance Hb 90–110 g L^−1^ for duration of hospital stay	Restrictive transfusion strategy initiated when Hb ≤75 g L^−1^, maintenance Hb 75–90 g L^−1^ for duration of hospital stay	Primary outcome: death and MACE at 30 days Secondary outcomes: myocardial injury, postoperative complications, transfusion requirements, discharge destination, duration of stay, HRQoL at 30 and 120 days
POP-I (in setup)	Adults aged ≥60 yrs with mild or moderate anaemia, Hb 80–110 g L^−1^, after emergency laparotomy or hip fracture surgery *n*=2400	Two comparator arms: i.v. FDM 20 mg kg^−1^ or i.v. FDM 20 mg kg^−1^ plus darbepoetin 2 mg kg^−1^ s.c.	Usual care control group	Primary outcome: DAH 30 Secondary outcomes: HRQoL at 30 and 120 days, DAH 120 days, mobility, residential status, duration of hospital stay, complications of treatment and mortality
ITACS NCT 02632760	Adults aged >18 yrs undergoing elective cardiac surgery with anaemia (haemoglobin males ≤130 g L^−1^, females ≤120 g L^−1^) *n*=1000	I.V. FCM or FDM 1000 mg, given 1–10 weeks before surgery	Placebo infusion	Primary outcome: DAH 90 Secondary outcomes: hospital and critical care length of stay, complications, Hb, blood transfusion, DAH 30, disability free survival, HRQoL

I.V. iron is a biologically plausible treatment option for postoperative anaemia. This approach is not currently supported by the available evidence base, although the CPOC guidance suggests that i.v. iron may help after surgery. Systematic reviews of postoperative iron therapy in elective surgery and major orthopaedic surgery (including hip fracture) have not provided conclusive evidence of an effect on clinically important or patient-centred outcomes.^32^, ^33^, ^34^ However, the included studies in these reviews were small with substantial clinical and methodological heterogeneity. Large trials investigating the clinical effectiveness of treating anaemia in patients undergoing hip fracture or emergency laparotomy surgery with i.v. iron with or without recombinant erythropoietin are underway (Table 2).

#### Safety considerations

Contemporary i.v. iron preparations have good safety profiles with a low incidence of adverse effects and anaphylaxis.^35^ Hypophosphataemia is a recognised complication of FCM and rare cases of hypophosphataemic osteomalacia and fractures requiring surgery have been reported. Recent guidance from the Medicines and Healthcare products Regulatory Agency (MHRA) recommends that phosphate concentrations should be monitored in patients who: (i) require multiple doses of FCM; (ii) are on long-term FCM treatment; and (iii) have certain risk factors (vitamin D deficiency, osteoporosis, inflammatory bowel disease, calcium and phosphate malabsorption). There is some evidence that this hypophosphataemia may be associated with slower recovery from fatigue. There is conflicting evidence about the risk of infection with i.v. iron. The available data are limited by the heterogeneity in the reporting of infection as an outcome measure and variability in transfusion practices. As discussed previously in this article, both iron deficiency and iron overload may be associated with an increased risk of infection. This risk may be become more pertinent in the acute postoperative phase and a careful risk–benefit assessment is required.^36^ Extravasation leading to lasting haemosiderin skin pigmentation has been observed and patients should be advised to report any pain/discomfort during the infusion.^37^

### Erythropoiesis-stimulating agents

Erythropoietin (EPO) is an essential hormone for RBC production. Perioperative inflammation can lead to decreased production of EPO and cause the bone marrow to become resistant to circulating EPO. Administration of erythropoiesis-stimulating agents (ESAs) stimulates EPO receptors and reduces hepcidin levels which in turn improves iron availability. Preclinical data show that ESAs may also have beneficial anti-inflammatory properties, especially on cytokines that are involved in iron restriction—IL-6, IL-1 and TNF-α.^38^

#### Clinical use

Clinical guidelines provide conflicting recommendations on the use of ESAs. CPOC guidelines suggest that preoperative treatment with ESAs should be considered in some specialist situations (Jehovah's witness, failure to respond to i.v. iron, renal failure).^3^ National Institute for Health and Care Excellence (NICE) guidance, albeit from 2015, recommends that EPO should not be used in surgical patients to reduce blood transfusion unless blood transfusion is refused, or compatible blood is unavailable.^39^ However, recent international recommendations support the use of ESAs for the preoperative treatment of anaemia, including in cardiac and orthopaedic surgery. If ESAs are used, then supplemental iron must be given.^40^^,^^41^ It is currently difficult to advocate the regular use of ESAs in UK perioperative practice without consideration of individualised care and discussion with expert clinicians more familiar with its use. Ongoing research will help define the optimal regimen for ESAs (Table 2).

#### Safety considerations

An increased risk of thromboembolic complications is a potential safety concern with use of ESAs. However, a recent systematic review found no difference in serious adverse events (mortality, stroke, pulmonary embolism, deep vein thrombosis, renal failure) in surgical patients who received ESAs and iron compared with iron alone.^42^ Previous reports of venous thromboembolism were observed in surgical patients not receiving thromboprophylaxis, which does not reflect current practice. The adverse effects of one single perioperative dose, rather than multiple doses—where the risk has been reported, also needs to be considered. Nevertheless, prophylactic mechanical and pharmacological thromboprophylaxis should be considered in all patients receiving perioperative ESAs.

### Blood transfusion

Blood transfusion is the direct replacement of blood to rapidly treat anaemia with the aim of immediately improving oxygen delivery to cells to prevent complications from hypovolaemia and organ hypoxia. Emergency transfusion will help to restore circulating volume and prevent complications of major haemorrhage: this will not be discussed here. In the preoperative setting, the appropriate approach to managing anaemia is to avoid blood transfusion. Transfusion does not treat the underlying pathology of anaemia caused by iron malabsorption, storage or availability.

#### Clinical use

Current consensus advocates a restrictive transfusion trigger in the perioperative period. This is supported by the NICE and NHS Blood and Transplant (NHSBT) recommendations for a restrictive blood transfusion threshold of Hb ≤70 g L^−1^ (with a target of 70–90 g L^−1^ after transfusion) in patients who do not have major haemorrhage, acute coronary syndrome or chronic anaemia requiring regular blood transfusions.^39^ There are some situations where restrictive transfusion strategies may not apply. In patients with chronic cardiovascular disease, evidence suggests higher rates of myocardial injury or infarction and a trend towards higher mortality in patients managed with restrictive transfusion strategies.^43^ This is an active area of research in hip fracture patients (Table 2). Data also remain insufficient to inform transfusion practice in other contexts such as acute neurological injury (including traumatic brain injury), stroke and haematological malignancy.^44^ There is also uncertainty in patients undergoing cancer surgery because of the hypothesised effects of transfusion on immunomodulation, particularly on tumour recurrence and postoperative infection.^45^

Pragmatically, decisions about blood transfusion are made on a case-by-case basis according to each patient's circumstances, accounting for the degree of anaemia, clinical impact of hypoxia, signs of organ ischaemia and the patient's comorbidities. These factors are balanced with their circulating volume status and pathophysiology. Other treatments for anaemia should also be considered at this juncture.

#### Safety considerations

The potential benefits of RBC transfusions must be considered alongside the risks. These include transfusion-related acute lung injury, transfusion associated circulatory overload, non-haemolytic febrile and haemolytic reactions and immunomodulation. Some of these have been mitigated with the widespread introduction of leukoreduction.

### Novel agents

New agents targeted towards the mechanisms that reverse iron restriction and reduce hepcidin concentrations are currently under development (Table 3). Prolyl hydroxylase inhibitors stimulate production of EPO, act on intestinal mucosa to increase iron absorption and lower hepcidin expression.

**Table 3:** Novel agents under development for the treatment of anaemia of inflammation. ACVR1, activin A receptor kinase 1; BMP, bone morphogenic protein; HIF, hypoxia inducible factor.

Agent	Mechanism of action	Stage of development
Monoclonal antibodies LY2787106 LY3113593 LY2928057	Neutralises hepcidin, prevents ferroportin internalisation Blocks BMP binding to its receptor which decreases hepcidin and increases iron Binds ferroportin blocking interactions with hepcidin allowing iron efflux	Phase I Phase I Phase I
Momelotinib	Blocks BMP receptor ACVR1 which inhibits hepatic hepcidin expression	Preclinical
Lexaptepid	Nucleotide that binds and inactivates hepcidin	Phase I
Prolyl hydroxylase inhibitors	HIF prolyl hydroxylase inhibitors that mimic natural response to hypoxia and decrease hepcidin	Phase III

## Conclusions

The persisting burden of perioperative iron deficiency anaemia suggests that current treatment pathways do not appear to be having a meaningful impact. Improvements in our understanding of the epidemiology and underlying mechanisms of iron deficiency offer the potential for considerable improvements. Ongoing clinical trials, powered to detect changes in patient-centred outcomes, will inform future clinical guidelines.

## Acknowledgements

We would like to thank Professor Hal Drakesmith's laboratory (Weatherall Institute of Molecular Medicine, University of Oxford) for providing Figure 1.

## Declaration of interests

AS is currently supported by a National Institute for Health and Care Research (NIHR) Academic Clinical Lectureship award and the NIHR Blood and Transplant Research Unit in Data Driven Transfusion-Practice (NIHR203334). AA's research department has received grant support from Syner-Med (UK), Vifor Pharma (Switzerland) and Pharmacosmos (Denmark). AA has received honoraria and travel support for consulting or lecturing from Ethicon Endosurgery (UK), Johnson & Johnson (UK), Olympus (UK) and Vifor Pharma (Switzerland). RCFS declares that they have no conflict of interest.

## MCQs

The associated MCQs (to support CME/CPD activity) will be accessible at www.bjaed.org/cme/home by subscribers to *BJA Education*.
